# Supplementary material for: Stereoscopic 3D geometric distortions analyzed from the viewer’s point of view
Source: PLoS One. 2020 Oct 15;15(10):e0240661. doi: 10.1371/journal.pone.0240661 (PMC7561172; doi:10.1371/journal.pone.0240661)
Supplement: Attachment — Submitted filename: Response to Reviewers_Final.pdf [file pone.0240661.s001.pdf]

# Response to Reviewers

## Manuscript ID.: PONE-D-20-04436
## Title: Stereoscopic 3D geometric distortions analyzed from the viewer's point of view

We thank the academic editor and reviewers for their constructive comments that helped to greatly improve the contents and presentations of this paper. We have revised the manuscript according to their suggestions.

Point-by-point responses to the comments are listed below.

We hope that the revised version of the manuscript and the responses in this letter are satisfactory. The Highlight changes of the manuscript can be found behind this response.

**(1) AE: The present work does not sufficiently extend beyond the authors' previous work, and does not provide enough analysis of the model and implications thereof (perhaps through simulations, if not experiments), but rather seems largely an exercise of explaining the math behind such a hypothetical analysis without following through on it.**

**Reviewer: The second major problem is that the results of the analysis appear trivial at least as far as they are presented in the manuscript. Eventually, the authors do not discuss what is the novelty of their results as compared to their or other authors' previous work in relation to the stated aims of the study.**

**Answer 1:** We newly add a discussion about the difference between Gao et al. [4] and this paper in the fourth paragraph of Discussion as follows:

*Gao et al. [4] proposed a geometric model for S3D and is the most related work. Gao et al. [4] analyzed the geometric distortions only based on the binocular depth cue and left a gap between geometric distortions and VIMS in S3D, i.e., the reason why geometric distortions may cause VIMS was not explicitly explained. This work bridges the gap by analyzing the depth-cue conflict and distinguishes from Gao et al. [4] in three aspects. First, we introduce a retinal disparity model to analyze geometric distortions in the eccentricity-disparity ( $Ec - D$ ) coordinates. The angular disparity ( $D$ ) represents the binocular depth cue and the visual eccentricity ( $Ec$ ) represents the monocular depth cues. As demonstrated in the horizontal and the vertical axis of Figs 4, 7, 9, 11, 14, the geometric distortions in terms of the monocular and the binocular are disentangled and can be discussed separately. Second, we simulate geometric distortions from the cyclopean eye to visually demonstrate the monocular perception, as illustrated in Figs 5, 6, 8, 10, 12, 13. Third and most importantly, with the help of the retinal disparity model and the visualization technique, we can see the inconsistency between the monocular and binocular depth cues to build a connection between geometric distortions and VIMS in S3D.*

**(2) It claims that the model simulates a broad range of monocular depth cues ("linear perspective, interposition (occlusion), object sizes, shades and shadows, texture gradients, accommodation and blur, aerial perspective, etc."), the only metric derived for their characterization is "eccentricity". No**

**explanation or justification is provided how this single metric can account for all the monocular cues listed. As it turns out, they in fact measure eccentricity on the retina of the imaginary cyclopean eye.**

**Answer 2:** We newly add a figure in Fig 2 as follows.

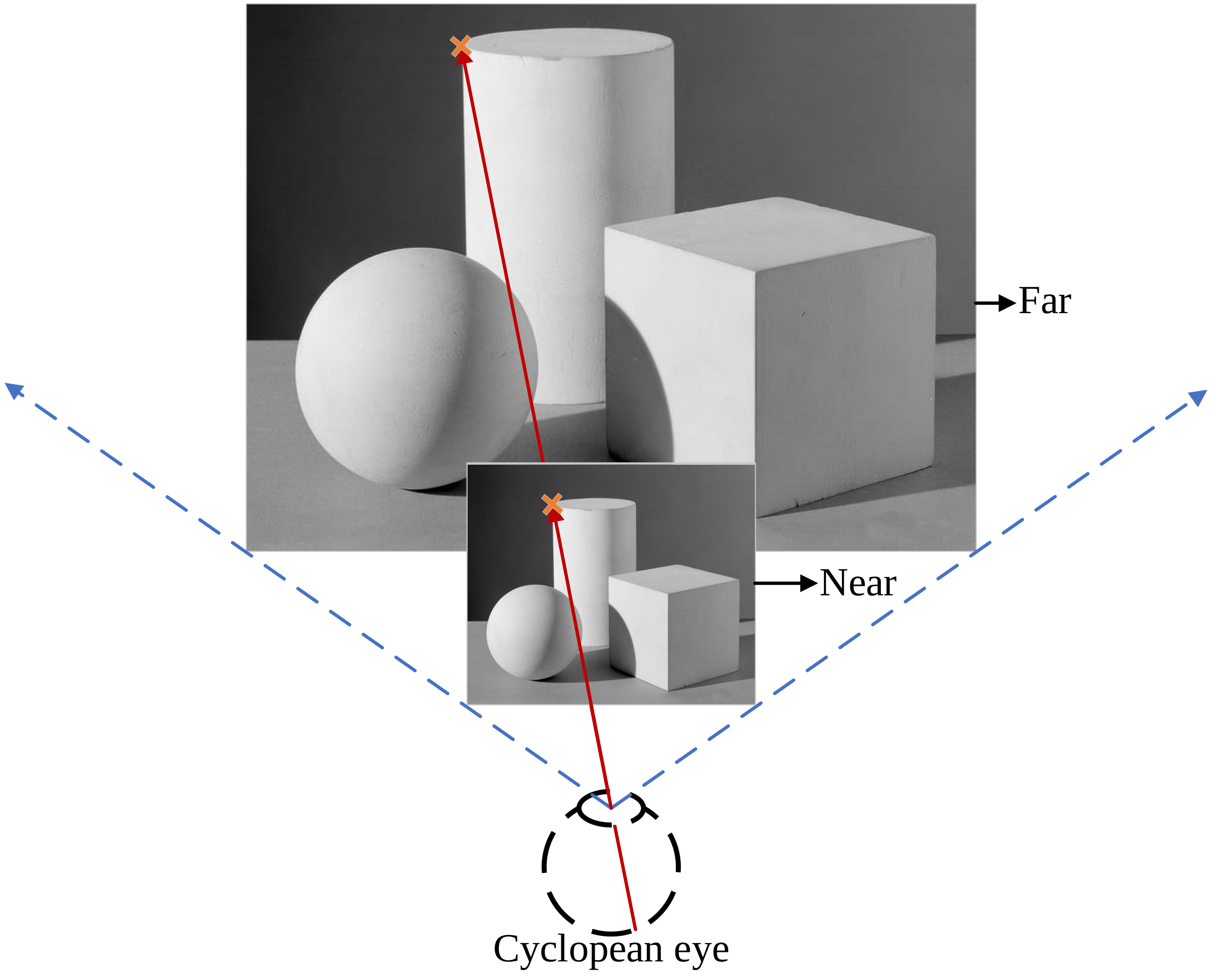


As illustrated in the figure, the far and the near objects project to the same retina locations of the cyclopean eye, including all the monocular depth cues (e.g., perspective, occlusion, shade and shadow, and texture gradient). If solely based on the visual eccentricity of the cyclopean eye, the far and the near objects are indistinguishable. Thus, the visual eccentricity (Ec) can account for all the monocular depth cues.

**(3) Indeed, except for a brief derivation of the effects of a simple horizontal head translation, no analysis of dynamic distortions is provided that might occur during scene motion.**

**Answer 3**: We newly added paragraph in Discussion as follows,

*Visually induced motion sickness (VIMS) involves motions. If the viewer watches a stationary S3D scene and stays still, geometric distortions with depth-cue conflicts may not cause any motion sickness symptoms. In a dynamic scene, for instance, when objects move towards the user in a distorted S3D space where the convergence distance is larger than screen distance (Section 3.2), monocular depth cues remain veridical while the binocular depth cue suggests objects at near or far distances seem to approach the viewer slower*

*or faster than the speed expected. Depth cue conflicts with motions may cause VIMS in S3D, which can be explained by the sensory rearrangement theory [9]*
